# Plectin in the Central Nervous System and a Putative Role in Brain Astrocytes

**DOI:** 10.3390/cells10092353

**Published:** 2021-09-08

**Authors:** Maja Potokar, Jernej Jorgačevski

**Affiliations:** 1 Laboratory of Neuroendocrinology-Molecular Cell Physiology, Institute of Pathophysiology, Faculty of Medicine, University of Ljubljana, Zaloška 4, 1000 Ljubljana, Slovenia; maja.potokar@mf.uni-lj.si; 2 Celica BIOMEDICAL, Tehnološki Park 24, 1000 Ljubljana, Slovenia

**Keywords:** plectin, central nervous system, astrocytes, glia, neurons, intermediate filaments, microtubules, actin filaments, cytolinker proteins

## Abstract

Plectin, a high-molecular-mass cytolinker, is abundantly expressed in the central nervous system (CNS). Currently, a limited amount of data about plectin in the CNS prevents us from seeing the complete picture of how plectin affects the functioning of the CNS as a whole. Yet, by analogy to its role in other tissues, it is anticipated that, in the CNS, plectin also functions as the key cytoskeleton interlinking molecule. Thus, it is likely involved in signalling processes, thereby affecting numerous fundamental functions in the brain and spinal cord. Versatile direct and indirect interactions of plectin with cytoskeletal filaments and enzymes in the cells of the CNS in normal physiological and in pathologic conditions remain to be fully addressed. Several pathologies of the CNS related to plectin have been discovered in patients with plectinopathies. However, in view of plectin as an integrator of a cohesive mesh of cellular proteins, it is important that the role of plectin is also considered in other CNS pathologies. This review summarizes the current knowledge of plectin in the CNS, focusing on plectin isoforms that have been detected in the CNS, along with its expression profile and distribution alongside diverse cytoskeleton filaments in CNS cell types. Considering that the bidirectional communication between neurons and glial cells, especially astrocytes, is crucial for proper functioning of the CNS, we place particular emphasis on the known roles of plectin in neurons, and we propose possible roles of plectin in astrocytes.

## 1. Plectin

Plectin is a large (>500 kDa) cytolinker, expressed in all mammalian tissues, and is especially abundant in epithelial and muscles cells and in the brain [1,2,3]. At the time of the discovery, when it was isolated as a high molecular weight polypeptide in rat glioma cell lysates [2], plectin was proposed to serve as a linker component between microtubules and intermediate filaments (IFs) [1,2]. However, in time, it was revealed that plectin is much more than merely a physical linker connecting cytoskeleton filaments [4]. Plectin provides a structural and signalling scaffold for cellular processes, ranging from cell adhesion and migration to mechanotransduction, organelle transport, and positioning, as shown in cell types of different tissues [4,5].

**Figure 1:**
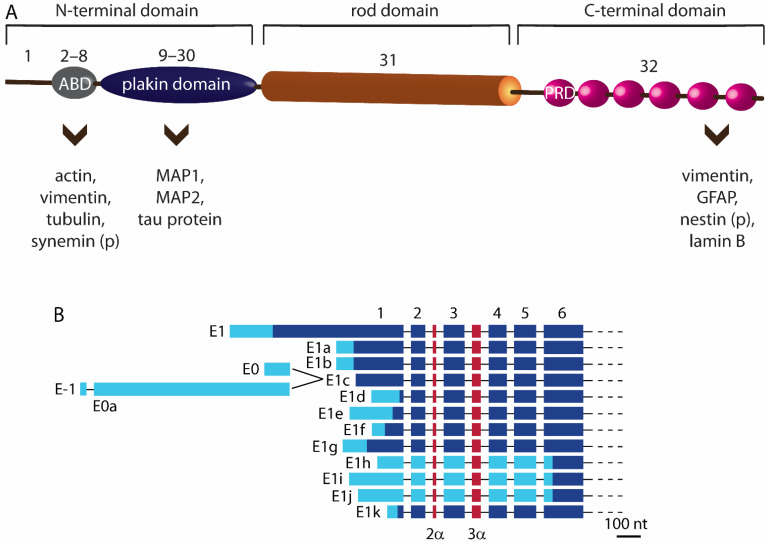
Schematic representation of plectin and its transcripts generated by alternative splicing of the 5’ end of the plectin gene *PLEC*. (**A**) The panel highlights interaction sites of cytoskeleton proteins and/or their associated proteins in astrocytes and neurons with their respective plectin domains. The C-terminal domain consists of six plectin repeat domains (PRD). (*p*), predicted interaction sites of certain IFs with plectin in astrocytes and neurons. Numbers above the schematic of plectin denote exons. ABD, actin binding domain. (**B**) Transcripts that give rise to individual plectin isoforms differ from each other only in short sequences at the 5’ end of the plectin gene. The numbers above the schematic denote consequent exons until exon 6. Exons 7 to 32 are not shown, as they are conserved among isoforms [4]. Individual exons are indicated by light and dark blue boxes, representing noncoding and coding regions, respectively. Red boxes denote two optionally spliced exons; 2α is inserted between exons 2 and 3, while 3α is inserted between exons 3 and 4.

The versatility of plectin is rooted in its structure, which enables binding of plectin to the building proteins of the cytoskeleton. The protein has a tripartite structure, comprising two large globular domains at each terminus connected by a central coiled coil of α helices [4]. All three domains of plectin participate in interactions with other proteins at their target sites (Figure 1A). The interaction is mediated either directly or indirectly. In the case of direct binding, plectin binds to all types of IFs, actin filaments and microtubules (possibly through isoform-specific plectin–microtubule binding). Alternatively, indirect binding of plectin is mediated by cytoskeleton-associated proteins (such as microtubule-associated proteins and myosin), including the building blocks of the subplasmalemmal cytoskeletal network, and other cellular proteins, such as the transmembrane receptors, components of the nuclear envelope, and several kinases [3,4,5,6]. Binding sites to different cellular proteins are found in the conserved parts of carboxy (C)- and amino (N)-terminal domains, such as the actin binding do-main (ABD) and plakin domain (Figure 1A), and are therefore preserved across all plectin isoforms [4]. Specifically, in astrocytes and neurons, ABD of plectin contains binding sites for actin, tubulin, IFs vimentin, and possibly synemin [4,7]. Microtubules interact with plectin also indirectly, via their associated proteins, MAP1, MAP2, and tau at the plakin domain [4]. Central α-helical coiled-coil rod domain of plectin mediates self-association [4]. C-terminal domain, consisting of six plectin repeat domains (PRD), harbours interaction sites for IFs vimentin, GFAP, lamin B, and probably for nestin [3,4,8]. C-terminal domain interactions are extensively regulated by reversible phosphorylation [9,10,11].

The tripartite structure of plectin is preserved in all basic plectin isoforms. Namely, plectin’s first alternative exons give rise to 12 basic isoforms, which differ from each other only in the short 5′-end structure of the protein (Figure 1B) [4]. The astonishing complexity of plectin isoforms is critically important for their tissue- and cell-specific targeting [4]. The variability of plectin isoforms is further enhanced by alternative splicing of the first non-coding exons and two short exons (2α, 3α) of ABD, some of them occurring specifically in neuronal tissue [4,12,13]. Apart from variable N termini, plectin isoforms also increase their variety through the lack of expression of exon 31, which encodes the rod domain. These rodless isoforms are expressed in different mouse, rat, and human tissues (skin, heart, brain, muscles, testis, liver), and have also been detected in rat glioma C6 cells [14,15,16,17,18,19].

Mutations in *PLEC*, a human plectin gene, cause severe malformations, known as plectinopathies, which include several types of epidermolysis bullosa simplex (EBS): EBS with muscular dystrophy (EBS-MD), EBS with a myasthenic syndrome (EBS-MD-MyS), EBS with pyloric atresia (EBS-PA), EBS-Ogna, EBS with nail dystrophy (EBSND), and limb-girdle muscular dystrophy (LGMDR17). Clinical manifestations of these malformations include muscular dystrophy, skin blistering, and also signs of neuropathy. Plectinopathies differ in affected tissues and organs, as well as mutations of specific plectin isoforms (for the detailed overview of symptoms and the list of mutations, please see [20,21]). In general, mutations of *PLEC* result in either markedly reduced or completely abolished plectin expression or by expressing only rodless plectin variants, as demonstrated in EBS-MD skeletal muscles [22]. Plectin mutations in EBS-MD patients and their links on CNS abnormalities are described below in the chapter Plectin-related Pathologies of the Central Nervous System.

## 2. Plectin in the Central Nervous System

**Figure 2:**
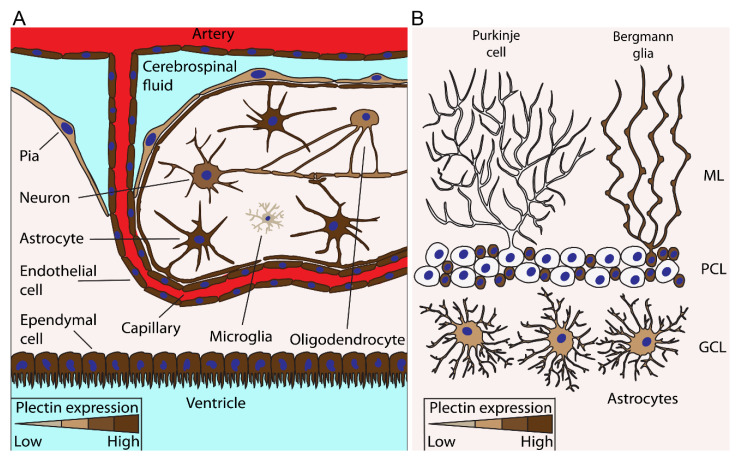
Expression of plectin in various cells types in the brain. The figure depicts plectin-expressing cells in the cerebrum (**A**) and cerebellum (**B**). (**A**), Particularly the abundant expression of plectin (dark brown) is a trait of cells such as astrocytes, endothelial cells, and ependymal cells at the boundaries between brain parenchyma and fluid-filled compartments; for example, arteries, capillaries, and ventricles. Besides cells at the border of the parenchyma and fluid-filled parts of the brain, neurons, oligodendrocytes, and intermittently microglia also express plectin. (**B**), In the cerebellum, Bergmann glia in the molecular layer (ML) and astrocytes in the granule cell layer (GCL) also express plectin; however, plectin was not detected in Purkinje cells from rat cerebellum [23]. The abundance of plectin expression in each particular cell type is denoted by the colour intensity, according to the colour scales at the bottom of each panel. PCL, Purkinje cell layer.

The presence of plectin throughout the central nervous system (CNS) was first confirmed by immunofluorescence staining, and specific regions of the adult rat CNS (telencephalon, diencephalon, cerebellum, brainstem, spinal cord, choroid plexus, and endothelial cells of blood vessels) were systematically screened [23]. Positive immunoreactivity for plectin was observed in ependymal cells, including the choroidal epithelial cells and tanycytes, astrocytes in the granule cell layer, grey matter, white matter and hypothalamus, Bergmann glial processes, radially oriented glial cells in the spinal cord, certain endothelial cells, and neurons [23,24]. Plectin is predominantly present in cells at the inner ventricular boundaries of the CNS, as well as at pia/glia and endothelia/glia junctional regions; however, it is expressed in practically all cell types of the CNS (Figure 2) at least in certain developmental or physiological stages [23,25]. Therefore, plectin is proposed to play a significant role in the maintenance of the aforementioned junctional regions, and the expression of defective plectin has been shown to alter the structural or functional integrity of the blood–brain barrier and of the pial surface [23,25].

### Plectin Isoforms and Rodless Plectin Variants in the Central Nervous System

In the brain, the P1c isoform is the most abundant, followed by P1e, P1g, and P1, while P1a, P1b, and P1f are only faintly expressed [18]. Although P1c shows a broad expression pattern over different tissues, a specific alternatively spliced variant composed of combinations of exons 1c, 2, 2α, and 3, and is expressed exclusively in the brain [18]. Exon 2α introduces additional short sequences in otherwise highly conserved ABD, leading to significantly enhanced binding capacity to actin, as observed in the skeletal muscle [18]. By analogy, another brain-specific isoform of plectin, i.e., transcript plec (0?,1c,2α,3α) containing the combination of unknown exon 0 that precedes exons 1c, 2α, and 3α, is expected to display distinctive plectin-binding characteristics to actin in the brain [18]. The untranslated nucleotide sequence of exon 0 in this isoform may lead plectin mRNA targeting in neurons or glia cells [18]. However, the localization of this isoform needs to be validated in distinct CNS cell types.

In general, the expression of rodless plectin variants in neuronal tissues is ~20-fold lower in comparison with their full-length counterparts; the P1c rodless variant is apparently the most abundant [17]. The function of rodless plectin remains an enigma in the CNS as well as in other tissues. For example, based on results obtained from a human keratinocyte cell line that lacks the full-length plectin, but still expresses the rodless variant in hemidesmosomes, it is hypothesized that the expression of rodless plectin might alleviate the symptoms arising from plectin insufficiency, i.e., compared with plectin-null mice with the lethal phenotype [26]. Then again, rodless mutation encoded by a nonsense mutation in *PLEC* exon 31 and an identical frameshift mutation in exon 32 in two patients with epidermolysis bullosa simplex (EBS) with a myasthenic syndrome (EBS-Mys) still showed a severe phenotype, which could not be explained solely by the rodless versus full transcript ratio when compared with control subjects, although a higher ratio was detected in the more affected patient [27]. In addition to the CNS, the expression of a rodless plectin isoform was also confirmed in neurons of the peripheral nervous system. The first confirmation came from a study of the rat superior cervical ganglion (SCG) [19]. The rodless plectin isoform encoding exons 1c-2-2α-3 was found to be upregulated in the postnatal stage, which supports the conclusion that rodless plectin may play a significant role in neuronal development [19]. The coiled-coil structure of a rod domain, which enables the formation of plectin oligomers, may impair the intracellular localization of plectin molecules and consequently affect cell adhesion and the mechanical, metabolic, and signalling properties of CNS cells.

## 3. Plectin in Specific Cell Types in the Central Nervous System

Plectin is a quintessential component of the cytoskeleton meshwork. On the one hand, it connects to microtubules and actin filaments that affect vesicle trafficking and organelle positioning [5,28,29], and on the other hand, it links extensively to IFs, which have multiple structural functions and modulate signal transduction in the CNS [30,31,32,33,34,35,36,37,38,39]. Thus, plectin is crucially positioned to fine-tune a myriad of cellular functions in cells of the CNS. Namely, each cell type in the CNS and/or their precursor cell expresses a unique set of IFs that varies in different pathophysiological settings and during development. Similar to the variability in the expression of a specific set of IFs in specific CNS cells, the expression pattern of plectin also changes at various developmental stages [12,19,40]. Although physical interactions between plectin and specific IFs in the cells of the CNS have been well established (Table 1), the physiological context of these interactions remains largely unexplored.

### 3.1. Plectin in Astrocytes

Astrocytes stand out among the cell types in the CNS, because they exert many functions that are fundamental for normal brain development and adult physiology, as well as contribute to specific CNS disorders. Besides their role in organizing the structural architecture of the brain parenchyma, such as demarcating the cortical and subcortical regions into functional compartments, astrocytes also interact with pia and the vasculature in a gliovascular network [45,46]. Moreover, astrocytes are intricately integrated into the stability and physiology of neural networks, governing neurogenesis, synaptogenesis, and synaptic activity by regulating ion, water, and metabolite homeostasis of the CNS [46,47,48,49,50]. In addition, the glymphatic system of perivascular tunnels formed by astroglial cells facilitates the flow of several compounds, including glucose, lipids, amino acids, growth factors, and neuromodulators around the brain and eliminates potentially neurotoxic waste products [51,52]. Being implicated at so many levels of CNS functioning, astrocyte dysfunction contributes to the pathogenesis of many if not all neurological disorders at primary or secondary levels [53]. Regardless of the level at which astrocytes contribute to neuropathology, this occurs through multiple and complex pathways ranging from a reactive astroglial response to astrodegeneration to pathological remodelling where proper functioning is either lost or modified [37,49,53].

#### 3.1.1. Interactions between Plectin and IFs in Astrocytes

In addition to microtubule and actin filaments, the astrocyte cytoskeleton consists of five types of IFs, i.e., glial fibrillary acidic protein (GFAP), vimentin, nestin, synemin, and lamin, the expression of which varies during development and physiological states of cells [12]. Astrocyte functions that are currently linked to the IF cytoskeleton have mainly been addressed from the perspective of particular types of IF. In contrast to the initial view of IFs as rather static elements, IFs facilitate various cellular activities and have been recognized as crucial in learning, memory formation, neurogenesis, as well as in glial scar formation and recovery after trauma. IFs are the most diverse group of cytoskeletal elements and are emerging as important integrators of various cellular processes [12], and also serve as arguably the most important binding partners of plectin; we focus here primarily on plectin–IF interactions.

In glial cells, the interaction between plectin and IFs was first observed in rat glioma C6 cells, where the distribution of plectin was detected along vimentin filaments, and extensive crosslinking between plectin and reconstituted vimentin filaments was corroborated in vitro [41]. Inter-bridging between plectin and vimentin, as well as between plectin and lamin B, is specifically regulated by protein kinases A and C, and the phosphorylation process is crucial for many interactions with the C-terminal domain of plectin [4,9,44]. In embryonal rat astrocytes, interactions between vimentin filaments and plectin were demonstrated only recently; plectin has been proposed to affect the migration process of astrocytes in mouse embryo [43]. Interactions between plectin and GFAP, which frequently forms co-polymers with vimentin, have been proposed to also play a role in collective migration of astrocytes during development [43], yet the exact mechanism remains to be further elucidated. Nevertheless, the first proof of binding between plectin and GFAP was demonstrated in extracts from the hog spinal cord [41] and was later confirmed also in primary human and neonatal mouse primary astrocytes [42]. Considering the recognized roles of vimentin and GFAP, such as in neurogenesis, myelinization, glial scar formation, maintenance and permeability of the blood–brain barrier, and response to intracellular and hypoosmotic stress [12], the impact of plectin–vimentin and plectin–GFAP interactions most likely extends to processes other than astrocyte motility.

Indications of plectin–vimentin and plectin–GFAP interactions in astrocytes are providing us with the first glimpse of the role of plectin–IF interactions in these cells, with a vast field of possible interactions between plectin and IFs (Table 1, Figure 1A) and their functional consequences to be explored. In contrast to vimentin and GFAP, for which the interactions with plectin have been shown in astrocytes, plectin–nestin interactions in astrocytes are not yet confirmed. In adult mouse CNS neurospheres, composed of neural stem or progenitor cells, phosphoproteome analysis identified a series of serines that represent specific phosphorylation sites in CNS nestin [8]. These serines also deserve further attention in astrocytes, because they might also be important for specific nestin interaction with plectin in this cell type. The role of nestin in neural stem cells was proposed to mediate the distribution of vimentin to daughter cells during self-renewal and neurogenesis [8]. Similarly, nestin might also affect the redistribution of certain IFs in astrocytes, especially considering that, in astrocytes, nestin has been correlated with the formation of astrocyte protrusions, motility, and notch signalling during hippocampal neurogenesis in cognitive functions [12,31,36]. If this is the case, it would be difficult to imagine that plectin, as the only plakin cytolinker in astrocytes, is excluded from these processes.

Similarly enigmatic to plectin–nestin interactions in astrocytes are interactions of plectin with synemin, which is expressed in astrocytomas as well as immature and reactive astrocytes, where it incorporates into vimentin filaments and associates with GFAP [54]. To date, physical linking between plectin and synemin has been demonstrated only in skeletal muscle, where plectin isoform P1 has been unambiguously shown to interact with β-synemin through three possible β-synemin-binding sites at the N-terminal end of plectin [7]. The P1 isoform is predominantly expressed in skeletal muscles, but it was also confirmed in the brain, albeit to a much lesser extent [18]. Nevertheless, synemin is predominantly expressed in immature and reactive astrocytes, i.e., in conditions in which the expression of the P1 isoform of plectin has not been verified. Hence, it remains to be verified if P1 isoform is up-regulated in parallel with synemin in the aforementioned settings. Alternatively, other CNS isoforms of plectin may also interact with synemin in astrocytes and further extend the functions of plectin.

#### 3.1.2. Interactions of Plectin with Microtubules and Actin Filaments in Astrocytes

Although the first proof that plectin interacts with microtubules was obtained by experiments performed on glioma C6 cell extracts, and soon after in brain microtubule preparations [11,29], the extent of plectin associations with microtubules in astrocytes remain to be investigated. C6 glioma cells were also instrumental in demonstrating the interaction between plectin and actin; i.e., immunogold labelling in C6 glioma cells was used to demonstrate that plectin molecules link IFs among themselves and with actin filaments [55]. The implication of plectin in the migration properties of astrocytes is therefore somehow anticipated, especially considering that plectin has been shown to also affect the morphological appearance of these cells. Namely, in primary mouse astrocytes, it was demonstrated that, through binding to actin filaments, plectin enables cells to acquire stellate morphology [56]. The role of plectin in astrocyte migration was assessed using siRNA, which silenced plectin expression in primary astrocytes and impeded their migration [43]. In a migrating monolayer of control astrocytes, focal adhesions (FAs), which co-localized with plectin and with IFs, were shown to be concentrated at the leading edge of leader cells [43]. De Pascalis and co-workers [43] suggested that P1f is the most probable candidate to mediate interactions between IFs and FAs in astrocytes. This assumption was based on the previous report on fibroblasts, where it has been shown that P1f is vital for docking vimentin filaments to mature FAs [57]. If this indeed is also the case in astrocytes remains to be investigated. In astrocytoma cells, high migration potential was correlated with the presence of another IF, i.e., synemin, at the leading edge; when synemin was downregulated, cell migration slowed down [58]. Considering that the P1 isoform binds to synemin in skeletal muscle [7], P1 isoform is a possible candidate that may contribute to migration of CNS cells in different physiological settings. However, FAs are not just an important factor in cell migration. FAs are specialized sites within the cell where clustered integrin receptors interact with the extracellular matrix; they also coordinate function for the entire cytoskeleton and act as scaffolds for many signalling pathways [59]. Drawing a parallel from previous studies on other cell types, such as fibroblasts and endothelial cells [56,57,60,61], we assume that plectin may affect several functions that are mediated by FAs.

#### 3.1.3. Presumed Roles of Plectin in Astrocytes

**Figure 3:**
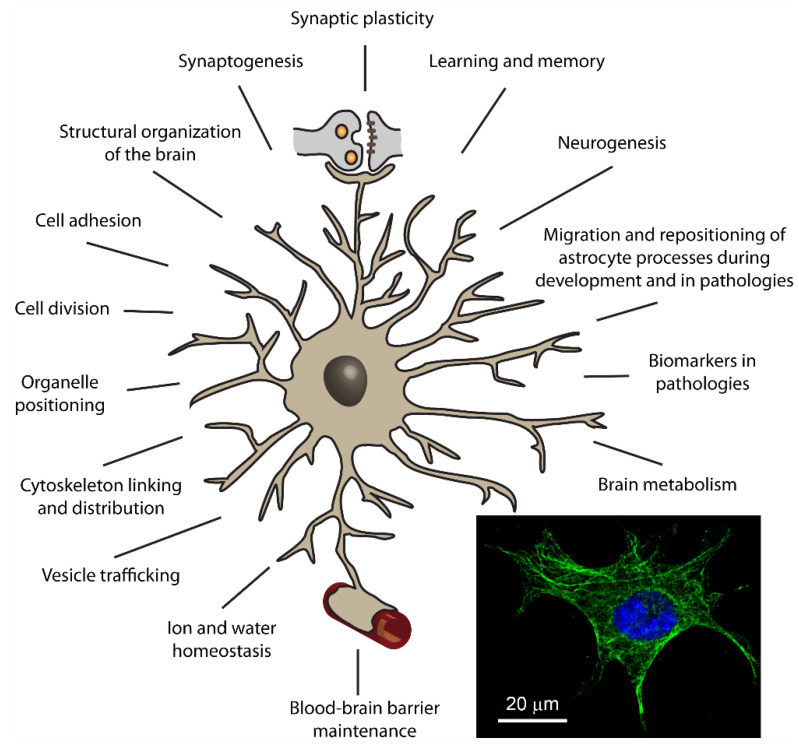
Putative roles of plectin in astrocytes. The figure depicts an astrocyte, a type of glial cell that, on the one hand, together with the pre- and postsynaptic membrane constitute the so-called tripartite synapse (depicted at the top of the figure), and on other hand, enwrap brain capillaries (indicated at the bottom of the figure). Presumed roles of plectin in astrocytes are summarized around the astrocyte. Based on today’s knowledge of plectin, derived from other cell types and biochemical studies, and cytoskeleton elements in the CNS, we propose that plectin plays crucial roles in a number of intracellular processes of astrocytes, which are also related to the functioning of other brain cells, such as endothelial cells and neurons. By affecting tissue morphology and physiology, plectin in astrocytes may have a prominent impact on higher order functions of the CNS, such as learning and memory formation. Inset: image of a mouse astrocyte in culture with immunolabelled plectin (green labelling) and DAPI-labelled cell nucleus (in blue). Note that the plectin signal results in a pronounced filamentous distribution, which likely denotes binding to the cytoskeleton.

As the cytoskeleton underlies cell mechanics, morphology, and physiology, and is thus implicated in basically all cellular functions, one can anticipate that molecules that are essential for its cellular positioning contribute a great deal to cytoskeleton-influenced cellular functions. Considering that plectin is the only member of the plakin family expressed in astrocytes [4,12], plectin likely has a key role in all types of cytoskeleton-dependent cellular processes in astrocytes, as depicted in Figure 3. One of the most important cytoskeleton-related processes is vesicle trafficking, which directs molecule uptake or discharge, organelle and mitotic spindle positioning, plasma membrane insertion of receptors and transporters, and cell-to-cell signalling [30,32,33,34,35,36,37,53,62,63,64]. Plectin-linked vesicle trafficking of intracellular molecules is also linked to cell metabolism. For example, in plectin/dystrophin double-deficient mice, impaired glucose uptake was linked to increased accumulation of P1f at the sarcolemma, which is the same condition as is found in Duchenne muscular dystrophy patients [65]. Impaired glucose uptake is the consequence of destabilized microtubule networks; i.e., microtubule-dependent vesicle transport of glucose transporter 4 from the cytoplasm to the sarcolemma is hindered [65]. However, the effect of plectin on cell metabolism is not unidirectional, since metabolism can also affect plectin levels. Namely, metabolic fuel has been shown to reduce the expression of plectin in primary rat astrocytes fuelled with hybrid fuels D-glucose and L-lactate, as opposed to astrocytes fueled with either fuel alone [66]. In addition, acute changes in metabolic fuel, or lack thereof, changed levels of clathrin and dynein, both involved in secretory vesicles trafficking and recycling [66]. Thus, plectin-related metabolic changes in astrocytes could be a significant contributor to neuropathologies. Namely, the metabolic and cytoskeletal changes in astrocytes may, through astrocyte–neuron metabolic cross-talk, significantly contribute to the course of inflammatory signaling, neurodegenerative diseases, and ageing [50,67]. On the cellular level, the key organelle involved in the regulation of cellular metabolism are mitochondria [68]. Deformed mitochondrial networks in terms of shape, subcellular distribution, and respiratory chain dysfunction have already been detected in skeletal muscles of EBD-MD patients [22,69,70].

To summarize, the diversity of already established and conceivable interactions between various cytoskeleton elements and plectin in astrocytes, combined with the versatile nature of plectin, implies that, in astrocytes, plectin is most likely involved in a broad selection of cellular processes, specified in Figure 3.

### 3.2. Plectin in Neurons

Knowledge of particular cytolinker functions (i.e., BPAG1, microtubule-actin crosslinking factor 1 [MACF1], actin crosslinking family 7 [ACF7] protein, and plectin) in neural cells of vertebrates is limited [71]. In addition to microtubules and actin filaments, neurons express four types of IFs: neurofilaments (NFs), α-internexin, peripherin, and nuclear lamins; the set of proteins vary between different types of neurons [23]. The interaction of plectin with NFs was first demonstrated in extracts from hog spinal cord [41]. This finding was subsequently confirmed in situ in a subpopulation of motoneurons of the adult rat brainstem and spinal cord where plectin abundantly co-localized with NFs in a population of motoneurons in the brainstem and spinal cord [23]. The same study described co-localization of plectin with peripherin in motoneurons of the rat brainstem and spinal cord.

To date, only P1c, which is the most abundant plectin isoform in neural cells, has been addressed in terms of its expression levels, combined with morphological and physiological implications in neurons. The latter was made possible by the generation of two knockout mouse lines; the one in which P1c was selectively lacking (P1c^−/−^), and the one in which plectin was conditionally deleted in neural cells and neuronal precursor cells [71]. The expression of plectin normally begins in the embryonal stage and is significantly boosted immediately after birth, followed by a steady increase with age, as demonstrated in mice whole-brain extracts [71]. P1c and rodless P1c generally follow such a developmentally regulated pattern of expression, with the exception that they were not detected in the embryonal stage [71]. Moreover, the ratio of expressed rodless P1c to the full-length P1c decreases with age [71], and low levels of rodless pan-plectin were also detected in MD-EBS keratinocytes [26].

In the cerebral cortex of the adult mouse brain, plectin exhibits widespread punctuate expression, exposing the P1c isoform as dominant in all cortical layers, whereas, in cells around the fourth ventricle, other isoforms are also prominent [71]. In postsynaptic dendrites of the brain cortex, P1c either co-localizes with MAP2 or is present in close proximity to MAP2 and synaptophysin, which is an integral membrane protein of synaptic vesicles. Together with the P1c expression pattern, these results implied that P1c could play a role in the universal formation of synapses and/or maintenance of their functional integrity, affecting behaviour and cognition [71]. A recent study [28] corroborated this hypothesis by showing that P1c affects neuritogenesis, neurite branching, and growth cone morphology in hippocampal neurons and in dorsal root ganglion neurons. Furthermore, P1c deficiency impaired the mobility and the directionality of synaptic vesicles and mitochondria, which was attributed to increased tau protein association and altered dynamics of neuronal microtubules in neurons of P1c^−/−^ mice [28]. Tau proteins, which are the only MAPs that exhibit an increase in microtubule association in the absence of P1c (which binds to tau proteins but can also bind directly to microtubules and is therefore a MAP per se), have been shown to spatially modulate the intracellular trafficking of vesicles and organelles [72,73]. Specifically, tau proteins act as an obstacle on the microtubules that impairs kinesin-driven transport and, to a lesser extent, also dynein-driven transport [72,73]. In agreement, P1c deficiency in neural cells attenuated primarily the speed of anterograde vesicles [28]. Deficits in vesicular transport in neurons, which is crucial for proper neuronal function and survival, have been associated with neurodevelopmental or neurodegenerative disorders, including amyotrophic lateral sclerosis, Alzheimer’s disease, and Huntington’s disease [74,75]. On the same note, P1c^−/−^ mice with altered vesicle trafficking exhibited impaired pain sensitivity, diminished learning capabilities, and reduced long-term memory [28]. Although there is no doubt that impaired vesicle mobility is high on the list of possible causes of the observed phenotype, other possibilities cannot be ruled out. Among others, plectin has been shown to bind several signalling molecules expressed in neurons, such as the NR3A subunit-containing N-methyl-D-aspartate (NMDA) receptors [76], the C-X-C chemokine receptor type 4 (CXCR-4) [77,78], the γ-subunit of AMP-activated protein kinase (AMPK) [79], receptor for activated C kinase 1 (RACK1) [80], and the tyrosine kinase Fer [81]. These signalling molecules have an important role in normal physiological conditions during neural development and in the mature brain where they modulate synaptic function and neuronal survival; however, they also contribute to the pathophysiology of various CNS diseases. To date, the aforementioned interactions of plectin with signalling molecules have been assessed exclusively in non-neural cells, such as dermal fibroblasts, differentiated myotubes, and keratinocytes. Therefore, future studies investigating the involvement of plectin in signalling cascades in neurons are required.

### 3.3. Expression of Plectin in Ependymal Cells, Tanycytes, Oligodendrocytes, Bergmann Glia, Microglia

A number of cell types in the CNS have not been investigated in studies directed specifically to the role of plectin and its co-distribution with respective sets of cytoskeletal elements that are expressed in these cell types. Ependymal cells, Bergmann glia, oligodendrocytes, and microglia have been mentioned in the context of plectin in only a handful of studies.

Ependymal cells in all brain ventricular regions and in the central canal of the spinal cord express plectin, which is especially abundant at the plasma membrane and to a lesser extent in the cytoplasm [23]. The scarcity of experimentally verified plectin isoforms in ependymal calls, which are for now limited to the P1c isoform, makes the prediction of possible functions of plectin in this cell type rather difficult. In tanycytes, which are a subtype of ependymal cells aligning the ventral portion of the third ventricle, plectin is expressed at the periphery of the cell body and in processes [23]. Apart from one report by Errante and co-workers [23], not much is known about the role of plectin in tanycytes, or indeed in ependymal cells in general. Ependymal cells are ciliated cells and the beating of their cilia, which contributes to the flow of cerebrospinal fluid that is essential for brain homoeostasis and functions, depends on the actin network [82]. Considering the involvement of plectin in the regulation of actin dynamics via plectin’s ABD [56], plectin is expectedly fundamental to the proper functioning of ependymal cells. In addition, ependymal cells are similar to mucosal epithelial cells with regard to the ciliated structure and columnar shape. Some forms of EBS, such as EBS with muscular dystrophy (EBS-MD), are also characterized by mucosal lesions involving the upper aerodigestive tract [83]. It would be interesting to learn of possible abnormalities in ependymal cells of patients with EBS, especially because this cell type has been shown to also express cytokeratins 5 and 8 (albeit in low levels) [84].

The first indication that oligodendrocytes may express plectin came from the observation of plectin immunoreactivity in glial fibres traversing the white matter in the spinal cord [23]. However, no detailed study about plectin expression in oligodendrocytes has been conducted. On the other hand, Schwann cells, myelinating cells in the peripheral nervous system (PNS), express plectin, in particular P1 and P1c [85]. The importance of plectin in the PNS has been demonstrated through concomitant myelin sheath deformations, coinciding with abrogated association of the dystroglycan complex with the IFs in Schwann cells lacking plectin [85]. In contrast to mature Schwann cells, mature oligodendrocytes are devoid of IFs; however, oligodendrocyte precursor cells (OPCs) express nestin and vimentin [86,87]. Therefore, plectin may play an important role during differentiation of OPCs into oligodendrocytes and in myelin sheet stability. OPCs in the adult brain are important for mediating remyelination of demyelinating lesions [88]. Myelin formation by oligodendrocytes is regulated by dystroglycan receptors, and the absence of normal dystroglycan expression has been shown to impair oligodendrocyte differentiation and normal production of myelin-specific proteins [89]. Because plectin can interact directly with dystrophin and β-dystroglycan, as demonstrated in muscle fibres [90], plectin may affect myelin sheet stability by interactions with the β-dystroglycan complex.

Bergmann glial cells play important roles in the development and functioning of the cerebellum, not only by defining the cyto-architecture of distinct cerebellar zones but also by fine-tuning the activity of synapses [91,92]. The processes of these cells, which are localized in the molecular layer of the cerebellum (see Figure 2B), express plectin throughout their length, with particularly enhanced expression in the terminal endings of their processes [23]. As Bergmann glia cells express IFs, such as vimentin and GFAP [93,94], plectin is likely one of the molecules to interlink them. These cells are equipped with a huge collection of plasma membrane receptors through which they sense and react to cerebellar synaptic activity [91]. The fact that IFs show an intricate association with plasma membrane proteins, including receptors and adhesion molecules [95], the status of plectin as an IF linker renders this protein one of the key molecules on which research should be conducted. In the cerebellum, plectin is also expressed in astrocytes in the granule cell layer and white fibre tract layer [23], where the role of plectin also remains unknown.

Similarly, knowledge about the expression of plectin in microglia is also very elusive. Immunolabelling of the human neocortex and hippocampus did not reveal particular plectin immunoreactivity in microglial cells labelled by an antigen to the microglia-specific transmembrane glycoprotein known as CD68/KP1 [25]. Nevertheless, the potential expression of plectin in microglia, at least in the early stages of development and in various pathological conditions, deserves further attention. In mice treated with kainic acid (KA), which induces an acute seizure response and dramatic reactivity of hippocampal microglia, plectin was among the top 25 differentially expressed genes that were only observed in the KA-treated group [96]. Furthermore, activated microglia and a subpopulation of the microglia cells in the adult CNS in normal conditions (as described in the adult rat cerebral cortex) express nestin and vimentin IFs around the nucleus and in parts of the cell processes [97,98]. Considering the importance of plectin in interlinking IFs, one might expect at least time- and stimulus-dependent expression of plectin in microglia and/or their progenitors.

## 4. Plectin-Related Pathologies of the Central Nervous System

In certain neuropathologies, the redistribution and/or upregulation of plectin has been observed, yet the exact role of plectin in various neurological conditions remains to be clarified. For example, in Alexander disease (AxD), a rare and usually fatal neurodegenerative disorder that is associated with GFAP mutations [99], plectin in astrocytes accumulates in protein aggregates called Rosenthal fibres (RFs) [42]. RFs reside in the cytoplasm and comprise several proteins (e.g., GFAP, vimentin, synemin, plectin, ubiquitin, small stress proteins Hsp25, and αB-crystallin) [42,100,101,102]; however, the specifics of cellular malfunctioning that leads to RF protein conglomerations are not clear yet. Moreover, it is still debated whether RFs, a pathological hallmark of AxD, are toxic for the cell or if they have a protective role [100]. In the AxD brain, GFAP-expressing astrocytes show extensive plectin cytoplasmic redistribution, observed as a rim-shaped lining at the outer border of RFs [42]. In addition to changes in the intracellular distribution, an increase in the expression of the plectin gene was also detected in several pathological conditions. For instance, significantly increased plectin levels were detected in the brain of patients with AxD [42]. Similarly, it is suggested that, for many other neurodegenerative disorders, cytoplasmic inclusion bodies may not be the main cause of toxicity in the CNS but probably represent a cellular protective response, for example in Parkinson’s disease, Alzheimer’s disease, amyotrophic lateral sclerosis, and the polyglutamine diseases including Huntington’s disease and many others, as summarized by Ross and Poirier [103]. The localization and function of plectin in these disorders is unclear and should be investigated. In addition, upregulation of the plectin gene was detected in hippocampal reactive astrocytes in patients with epilepsy or Ammon’s horn sclerosis [25]. The upregulation of plectin, coinciding with the increase in GFAP levels, was even proposed to be a general feature when detecting the reactive astroglia [25]. Higher levels of plectin expression surely affect interlinking among the cytoskeleton components, and other proteins to which plectin adheres, which should be investigated in the future. Contrary to enhanced expression, the impaired expression of the plectin gene may also lead to the redistribution of various cytoskeleton elements in CNS cells. These examples are for now limited to animal models; for example, the absence of plectin in neonatal mouse astrocytes was shown to impair the organization of GFAP filaments, which was evident as an increase in the abundance of short-filament aggregates of GFAP and GFAP perinuclear bundles [42]. Similar to the absence of plectin, the most common AxD mutation (GFAP R239C) induces improper organization of the GFAP filamentous network [42].

In addition to the redistribution of plectin and alterations in the expression of the plectin gene in general, specific mutations of plectin isoforms need to be investigated in the brain, as well as in some other tissues. For instance, in patients with EBS plectinopathies, there is a robustly verified relationship between disruption of the normal keratin IF anchorage and hemidesmosomes leading to skin blistering [5]. In contrast, much less is known about the connection between plectin mutations and malformations in striated muscle [22], and only a few reports are available about malformations of the CNS and specific plectin mutations that may be directly linked to CNS pathologies. In all detected cases linked to patients with EBS, who typically exhibit skin and skeletal muscle malfunctions, malformations of the CNS were described infrequently. In EBS-MD patients with drastic disorganization of the myogenic IF cytoskeleton and severe mitochondrial dysfunction in muscle tissue, severe brain atrophy was also detected [69]. Screening for plectin mutation specifically in exons 31 and 32 revealed a homozygous 16-bp insertion mutation close to the IF-binding site in plectin C-terminal domain that results in a truncated plectin molecule [69]. Unfortunately, the link between plectin mutation and brain atrophy was not studied in detail, as the study focused on the irregularities observed in muscle tissue where lower expression of plectin was linked to severe desmin accumulations and mitochondrial abnormalities (abnormal positioning and structure with decreased enzyme activities) [69]. In skeletal muscle, the transcription levels of plectin mRNA remained unchanged (plectin isoform identity was not examined), but markedly depleted levels of the mutant plectin variant were observed, presumably due to premature decay of mutated plectin [69]. In a different study, computed tomography and magnetic resonance imaging revealed extensive cerebral and cerebellar atrophy, and screening revealed a homozygous 8-bp frameshift mutation in exon 31 (rod domain) that resulted in a premature termination codon [104]. Furthermore, a post-mortem examination of another patient with EBS-MD also revealed CNS pathology, which manifested as severe generalized atrophy of the cerebrum and cerebellum with increased gliosis combined with softening or loss of brain parenchyma; however, in this study, plectin mutation was not specified [105]. Neurodegeneration in patients with EBS-MD is possibly more widespread but remains underreported. Nonetheless, this is a rare condition (with approximately 40 cases to date; data collected at the portal for rare diseases and orphan drugs orpha.net), and accurate screening for CNS abnormalities is of the utmost importance. Increased screening of plectin mutations and its isoforms in patients with EBS and CNS deformations will reveal which mutations affect the CNS and will enable generation of tools for the study of mutant plectin-affected cellular processes in CNS cells.

Outside the CNS, some plectin isoforms have recently been revealed as biomarkers in pancreatic ductal adenocarcinoma, or potential biomarkers for several other types of cancer, i.e., human colorectal adenoma, lung cancer, head and neck squamous cell carcinoma, and non-metastatic oral squamous cell carcinoma [106,107,108,109,110]. Surprisingly, in the case of astrocytoma, the biomarker potential of plectin has not been thoroughly addressed yet.

## 5. Conclusions

Our knowledge of the roles of plectin in the CNS has been steadily increasing over the past few decades, but it still remains fragmental. Apart from the advancing research conducted on plectin in neurons and astrocytes, this field is still largely unexplored, especially if we consider other CNS cell types, such as ependymal cells, oligodendrocytes, and Bergmann glia, where plectin expression was identified, but its functions in these cell types await further research.

Here, we have summarized the current knowledge of plectin in the CNS and reviewed processes in which plectin is expected to be involved, particularly (but not exclusively) those that depend on the mechanical and signalling properties of IFs. One of the key challenges of future studies focusing on plectin in the CNS will be to account for the variability in the expression patterns of IFs and specific plectin isoforms in the individual cell types related to various developmental and pathological stages. To define its contribution to the functioning of the CNS, future studies will need to address possible roles of plectin in the CNS in connection with plectin’s interactions with distinct types of cytoskeleton, plasma membrane receptors, anchoring proteins, various organelles, extracellular matrix, and enzymes involved in intracellular signalling. In addition, mutations in the plectin gene, which have been identified in patients with CNS abnormalities, await further assessment. A potential role of plectin and its isoforms as a biomarker of CNS neoplasms should also be explored.

Considering the relative scarcity of data concerning the role of plectin in various CNS cell types, it seems all the more important to investigate plectin’s contribution in different physiological and pathological settings. Taking into consideration a wide spectrum of plectin’s known functions as an organizer of the cytoskeleton meshwork and regulator of cellular morphology and plasticity, there is no doubt that plectin deserves further attention from the neuroscience community.

## Figures and Tables

**Table 1:** Crosslinking and co-localization of plectin with cytoskeleton in cells and tissues of the central nervous system.

Cell/Tissue	Type of Cytoskeleton	Interaction Detected between Plectin and the Cytoskeleton	References
Hog spinal cord, astrocytes in rat brain, primary astrocytes (human, mouse)	Glial fibrillary acidic protein (GFAP)	Electron microscopy, in vitro; immunofluorescence, in situ; coimmunoprecipitation	[23,41,42]
Rat glioma C6 cell line, primary mouse astrocytes, white matter astrocytes and choroidal epithelial cells in rat brain, Bergmann glia in rat cerebellum	Vimentin	Electron microscopy, in situ and in vitro; immunofluorescence, in situ; coimmunoprecipitation; biochemical binding assays	[9,23,41,43,44]
Neurospheres generated from adult mouse forebrain	Nestin	Indirect proof; CNS nestin-specific phosphorylation sites detected by phosphoproteome analysis	[8]
Recombinant plectin P1	Synemin	Pull-down assays	[7]
Rat glioma C6 cell line	Lamin B	Biochemical binding assays	[9,44]
Rat motoneurons in brainstem and spinal cord	Peripherin	Immunofluorescence, in situ	[23]
Hog spinal cord, rat motoneurons in brainstem and spinal cord	Neurofilaments	Electron microscopy, in situ and in vitro; immunofluorescence, in situ	[23,41]
Rat glioma C6 cell line, dorsal root ganglion neurons, hippocampal neurons	Microtubules	Electron microscopy in vitro;immunofluorescence, in situ	[28,29]
Rat glioma C6 cell line	Actin filaments	Electron microscopy, in situ	[9]
